# Application of Lipid Class Ratios for Sample Stability Monitoring—Evaluation of Murine Tissue Homogenates and SDS as a Stabilizer

**DOI:** 10.3390/metabo11050277

**Published:** 2021-04-27

**Authors:** Sabrina Krautbauer, Raquel Blazquez, Gerhard Liebisch, Marcus Hoering, Philip Neubert, Tobias Pukrop, Ralph Burkhardt, Alexander Sigruener

**Affiliations:** 1 Institute of Clinical Chemistry and Laboratory Medicine, University Hospital Regensburg, Franz-Josef-Strauß-Allee 11, 93053 Regensburg, Germany; sabrina.krautbauer@ukr.de (S.K.); gerhard.liebisch@ukr.de (G.L.); marcus.hoering@ukr.de (M.H.); philip.neubert@stud.uni-regensburg.de (P.N.); ralph.burkhardt@ukr.de (R.B.); 2 Department of Internal Medicine III, University Hospital Regensburg, Franz-Josef-Strauß-Allee 11, 93053 Regensburg, Germany; raquel.blazquez@ukr.de (R.B.); tobias.pukrop@ukr.de (T.P.)

**Keywords:** lipidomics, mass spectrometry, stabilization, sodium dodecyl sulfate, lipolytic ratios, lipid class ratio, tissue

## Abstract

Lipids are a ubiquitous class of structurally complex molecules involved in various biological processes. In the fast-growing field of lipidomics, preanalytical issues are frequently neglected. Here, we investigated the stability of lipid profiles of murine liver, brain, lung, heart, and spleen homogenates by quantitative flow injection analysis using tandem mass spectrometry and high-resolution mass spectrometry. Storage of tissue homogenates at room temperature showed substantial alterations of the lipid profiles reflecting lipolytic action. Therefore, ratios of ceramide to sphingomyelin, lysophosphatidylethanolamine to phosphatidylethanolamine, lysophosphatidylcholine to phosphatidylcholine, and diglyceride to triglyceride were applied to monitor sample stability and the effect of sodium dodecyl sulfate (SDS) as a potential stabilizing agent. The addition of SDS led to a concentration-dependent stabilization of lipid profiles in liver, brain, and heart homogenates, while in lung and spleen homogenates, in particular, the lysophosphatidylethanolamine to phosphatidylethanolamine ratio increased upon addition of SDS. In conclusion, we demonstrated that lipid class ratios reflecting lipolytic activity could be applied to evaluate both the stability of samples and the influence of stabilizers.

## 1. Introduction

Lipids are a ubiquitous class of structurally complex molecules involved in various biological processes. Their role in health and disease is extensively studied. In the fast-growing field of lipidomics, the focus lies on development and improvement of analytical technologies and methods, along with bioinformatics approaches to handle the huge and complex data obtained. Although efforts to standardize lipidomic analyses and reported results are in progress, a broadly accepted consensus is still missing [1,2,3]. Different workflows for pre-analytical and analytical processes (e.g., sampling, handling, transport, storage conditions, sample preparation, and analysis) influence the stability and detection of lipids. This reduces comparability of results and complicates interpretation. Physical, chemical, and/or enzymatic reactions result in modification or degradation of lipids. To improve lipid stability, these reactions should be reduced, which is challenging due to the high diversity of lipid species and the various different sample types. A review summarizing the current knowledge about lipid stability and standardization efforts was recently published [4]. Commonly organic solvents, additives, heat treatments, keeping samples cold, and minimizing handling and storage times are recommended to quench enzymatic activity [4,5,6]. In addition, methods may need to be modified when samples from obese subjects are studied [7].

Sodium dodecyl sulfate (SDS) is an anionic surfactant commonly used as a component for lysing cells during nucleic acid extraction and for denaturing proteins [8]. SDS-induced unfolding of protein tertiary structure may interfere with catalytic activity and/or substrate binding of enzymes. Due to its characteristics, SDS may prevent enzymatic modification or degradation of lipids without interfering with their subsequent detection.

Here, we analyzed the effect of sample storage in methanol/water (50/50, *v/v*) at room temperature and compared the influence of different SDS concentrations on the stability of lipids in mouse liver, brain, lung, heart, and spleen.

## 2. Results

### 2.1. Alterations of Lipid Profiles in Murine Liver Homogenates Indicate Lipolytic Activity

**Figure 1:**
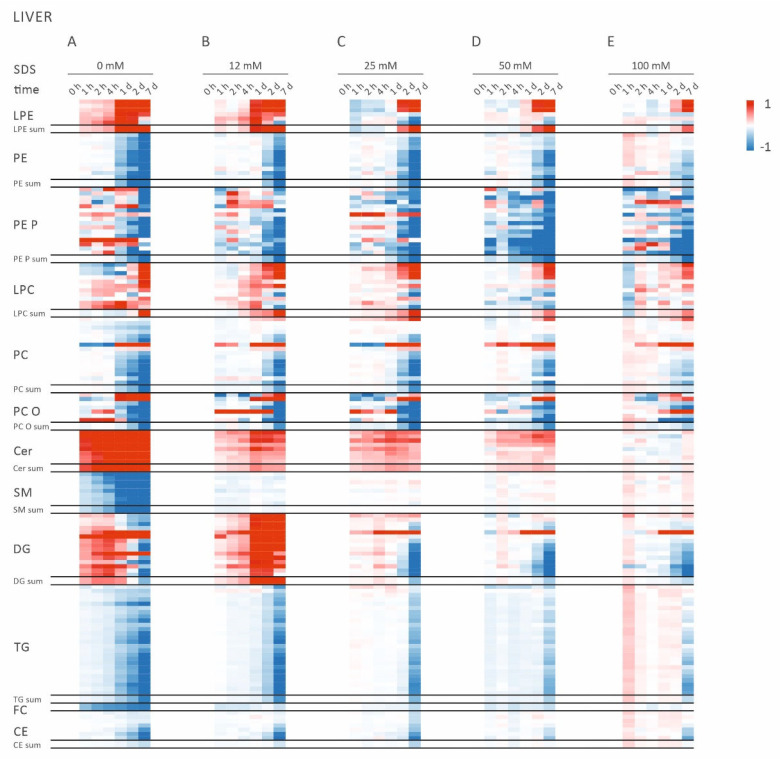
Heat maps showing the results of the lipidomic analysis of liver homogenates stored at room temperature for the indicated times. The color scale visualizes the log_2_-fold change relative to the control sample (0 h) of each group. Values <−1 were set to −1 and values >1 to 1. Samples were stored in methanol/water (50/50, *v/v*) without SDS (**A**) and with 12 mM SDS (**B**), 25 mM SDS (**C**), 50 mM SDS (**D**), or 100 mM SDS (**E**). CE: cholesteryl ester; Cer: ceramide; DG: diglyceride; FC: free cholesterol; LPC: lysophosphatidylcholine; LPE: lysophosphatidylethanolamine; PC: phosphatidylcholine; PC O: PC ether; PE: phosphatidylethanolamine; PE P: PE based plasmalogens; SDS: sodium dodecyl sulfate; SM: sphingomyelin; TG: triglyceride.

Lipid profiles of liver samples were determined by quantitative lipidomic analysis (Supplementary File Data S1), and heat map analyses were performed to gain an overview of the storage time-dependent alterations (Figure 1).

Storage of liver samples at room temperature altered lipid composition in a time-dependent manner (Figure 1A). Already after 1 h, the concentrations of all ceramide (Cer) species were elevated, whereas all sphingomyelin (SM) species were found to be decreased. These effects increased with prolonged storage at room temperature up to 7 days. Similar to Cer, increased lysophosphatidylethanolamine (LPE) and diglyceride (DG) species were observed after 1 h at room temperature. Interestingly, DG species were found to increase up to day 1 and decrease on day 2 and day 7. This may reflect further degradation to monoglycerides and fatty acids. No changes were observed for phosphatidylcholine (PC), phosphatidylethanolamine (PE), or triglyceride (TG) lipid species for up to 4 h. However, prolonged storage led to a reduction of these lipid classes. Taken together, storage of liver tissue homogenates in methanol/water (50/50, *v/v*) at room temperature revealed extensive changes in lipid composition, reflecting lipase activities.

### 2.2. Addition of SDS Stabilizes Lipid Profiles of Murin Liver Homogenates

Next, we tested whether the addition of SDS stabilizes lipid profiles of liver homogenates at room temperature (Figure 1B–E). We observed a concentration-dependent stabilization of the lipid profiles for up to 4 h. At least 25 mM SDS was necessary to prevent substantial generation of Cer, LPE, and DG. Higher concentrations up to 100 mM neither change lipid concentration nor affect mass spectrometric quantification.

### 2.3. Lipid Class Ratios Reflecting Lipolytic Activity Facilitate Easy Monitoring of Sample Stability

**Figure 2:**
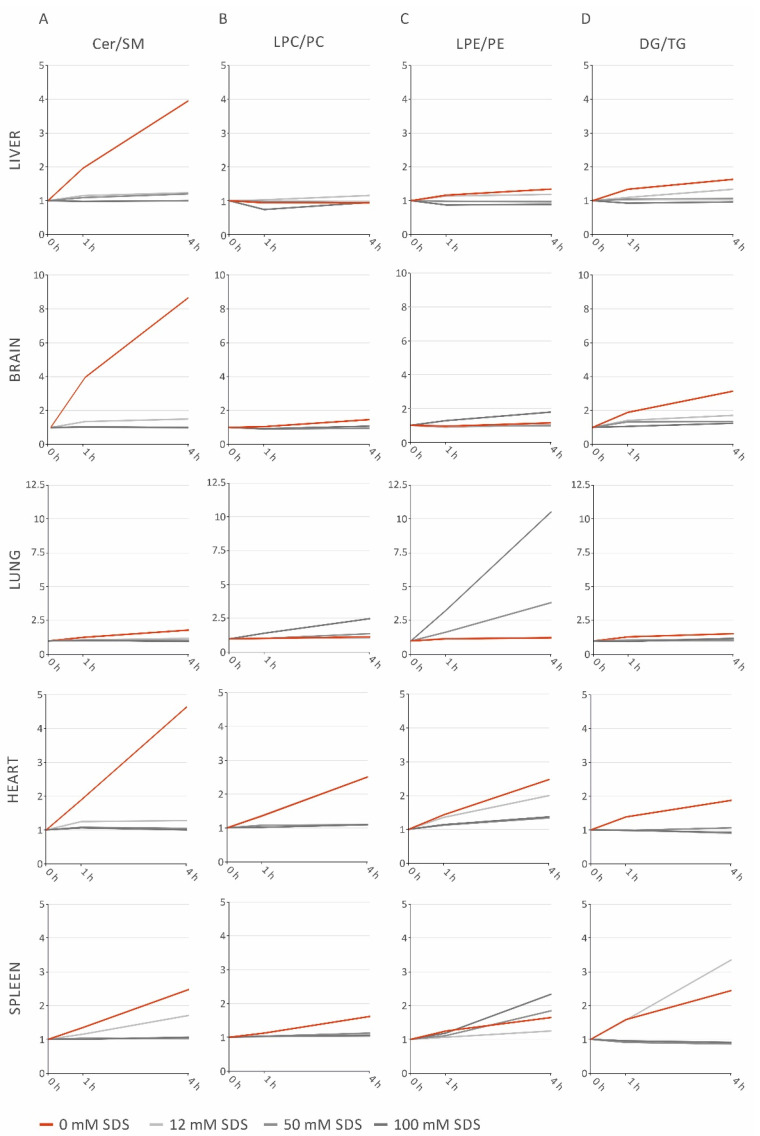
Lipid class ratios of liver, brain, lung, heart, and spleen samples stored at room temperature for indicated times. (**A**) Cer/SM ratios; (**B**) LPC/PC ratios; (**C**) LPE/PE ratios; (**D**) DG/TG ratios. The fold changes of the ratios are displayed relative to the control sample (0 h). Cer: ceramide; DG: diglyceride; LPC: lysophosphatidylcholine; LPE: lysophosphatidylethanolamine; PC: phosphatidylcholine; PE: phosphatidylethanolamine; SDS: sodium dodecyl sulfate; SM: sphingomyelin; TG: triglyceride.

Ratios of lipid classes reflecting the observed lipolytic activity were calculated to evaluate their use as sensitive degradation markers in various murine tissue homogenates. Tested ratios (lipid class sums) were Cer/SM, LPE/PE, DG/TG, LPC/PC, and free cholesterol (FC) to cholesteryl ester (CE). First, these ratios were investigated in liver homogenates, where the Cer/SM ratio increased during the first 4 h in the absence of SDS (Figure 2A). Compared to Cer/SM, the LPE/PE (Figure 2C) and DG/TG (Figure 2D) ratios showed only slight increases and the LPC/PC ratio was not affected up to 4 h (Figure 2B). No changes in the FC/CE ratio were observed (Supplementary Figure S1). 

Next, suitability of lipid ratios for stability monitoring was tested in brain, lung, heart, and spleen homogenates (Figure 2). As observed in liver, Cer/SM ratios revealed the most pronounced increase in all tissues without SDS stabilization. In general, tissues differ substantially in their lipolytic activity. Heart homogenate showed marked rises in all tested ratios, while lung samples were comparatively stable. In summary, lipolytic ratios facilitate an easy monitoring of sample stability as well as identification of the most instable lipid classes. Species profiles of lipolytic products displayed changes within a few hours (Supplementary File Data S1).

### 2.4. SDS Stabilizes Lipid Profiles of Brain and Heart Homogenates, but Increases LPE in Lung and Spleen Homogenates

**Figure 3:**
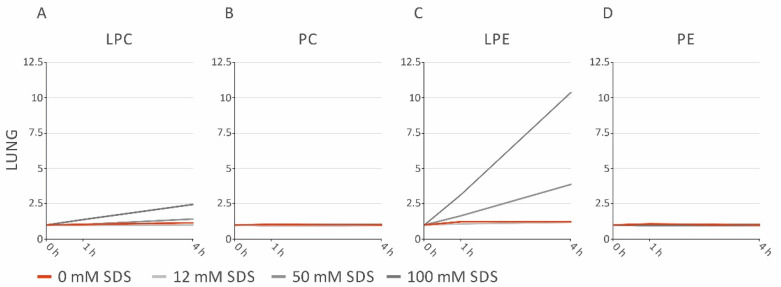
Fold changes of LPC, PC, LPE, and PE in lung samples stored at room temperature for indicated times. (**A**) LPC; (**B**) PC; (**C**) LPE; (**D**) PE. The fold changes are displayed relative to the control sample (0 h). LPC: lysophosphatidylcholine; LPE: lysophosphatidylethanolamine; PC: phosphatidylcholine; PE: phosphatidylethanolamine; SDS: sodium dodecyl sulfate.

Lipolytic ratios were applied to examine sample stabilization by SDS in brain, liver, lung, heart, and spleen samples. SDS concentrations ≥25 mM prevented a rise of the Cer/SM (Figure 2A) and DG/TG (Figure 2D) ratios in all investigated tissues up to 4 h (Figure 2A). The LPC/PC ratios in the brain, heart, and spleen (Figure 2B) were stabilized by SDS. In contrast, tissue-specific effects were observed upon SDS addition, concerning the LPE/PE ratios (Figure 2C); in liver and heart, PE degradation was prevented by SDS. However, the LPE/PE ratio increased at higher concentrations in spleen homogenates and was even more pronounced in lung homogenates. In addition, the LPC/PC ratio rose to higher SDS concentrations in lung homogenates. These findings were caused by SDS, since LPE and LPC increased depending on concentration, while PE and PC remained unchanged (Figure 3). No changes in the FC/CE ratio were observed (Supplementary Figure S1). Taken together, these data indicate a tissue-specific effect of SDS addition.

## 3. Discussion

Inappropriate preanalytic conditions may impair lipidomic data, for instance by lipolytic degradation [4]. Here, we investigated the stability of murine tissue homogenates prepared in methanol/water (50/50, *v/v*). Our data demonstrated that these samples were prone to rapid lipolysis despite high organic solvent content. In particular, a substantial increase of Cer, accompanied by a decrease of SM species, was observed after 1 h at room temperature in all investigated tissues. Cer/SM ratios were introduced to facilitate a simple monitoring of this conversion, which is most likely mediated by sphingomyelinases (SMase) [9,10]. Likewise, lipolytic degradation of other lipid classes was analyzed using ratios, i.e., LPC/PC, LPE/PE, and DG/TG. Tissue homogenates showed distinct lipolytic patterns, presumably related to their lipase expression. Of note, DG may be released not only by TG lipolysis but also by phospholipase C action from glycerophospholipids [4]. Therefore, the DG/TG ratio could be more appropriate to monitor TG-rich samples, such as adipose tissue or fatty liver.

In the next step, lipolytic ratios were applied to investigate whether SDS, as a protein denaturing agent [8], could be used to quench lipase activities in homogenized samples. Of note, SDS is known for its ionizability during electrospray ionization. Thus, introducing high amounts of SDS may increase suppression effects. This is an important factor, which should be considered. Here, we extracted lipids according to the procedure described by Bligh/Dyer, a chloroform-based method [11]. In general, SDS is relatively insoluble in chloroform but may transfer to the chloroform phase bound to other analytes or upon acidification. Nevertheless, we could detect SDS signals in both positive and negative ion modes. However, the rather low abundance did not affect sensitivity or lipid quantification up to the highest SDS concentration of 100 mM. The experiments were performed by FIA but derived from these data, and we did not expect any effect for LC-based methods. Carry over of SDS signals between samples was neglectable. While in all tissues SDS effectively prevented Cer and DG generation, a tissue-specific influence was observed on other lipid classes. Unexpectedly, LPE/PE in lung and spleen homogenates, as well as LPC/PC in lung homogenates, were increased by the addition of higher SDS concentrations. While the underlying reasons for this observation presently remain unknown, it may be speculated that lipases are activated by higher SDS concentrations. Additives like phenylmethylsulfonyl fluoride or heat treatment were previously shown to improve lipid stability by reducing enzymatic degradation. While additives work well in liquid samples, heat treatment can be used for solid samples [6,12]. This is especially true for invertebrate samples, in which heat treatment can be applied to the whole invertebrate. For mammalian samples, heat treatment may be applied after sampling [6]. This may still be an advantage if an appropriate device is available as stabilizers work after homogenization of solid samples, not reducing degradation up to that point. 

In summary, lipid class ratios provide a simple but powerful readout to monitor the stability of samples and evaluate the feasibility of stabilizing agents or conditions. In the investigated tissues, the Cer/SM ratio has been identified as the most sensitive marker to monitor lipolytic activity. Potentially, reference intervals of such lipolysis markers could help to assess the quality of preanalytic conditions, including snap freezing, storage temperature/time, and freeze thaw cycles. Furthermore, we demonstrated that the addition of SDS quenches enzymatic activity and can be used to preserve lipid stability during sample storage and handling. Of note, stabilization is lipid class and tissue-specific and needs to be evaluated respectively.

## 4. Materials and Methods

### 4.1. Chemicals and Lipid Standards

Chloroform and 2-propanol were purchased from Roth (Karlsruhe, Germany) and methanol from Merck (Darmstadt, Germany). All solvents were HPLC grade. Nuclease-free water was obtained from B. Braun (Melsungen, Germany). Ammonium formate, SDS, and CE standards were purchased from Sigma-Aldrich (Taufkirchen, Germany). [25,26,26,26,27,27,27-D_7_]-cholesterol was acquired from Cambridge Isotope Laboratories (Andover, MA, USA) with isotope purity higher than 98%. TG and DG standards were purchased from Larodan (Solna, Sweden). PC, Cer, SM, LPC, and LPE standards were purchased from Avanti Polar Lipids (Alabaster, AL, USA)

### 4.2. Animals and Tissue Harvesting

Procedures were complied with the German Law on Animal Protection and the Institute for Laboratory Animal Research Guide for the Care and Use of Laboratory Animals. The C57BL/6 mice (5 female, 6 male) used in this study were residuals within breeding. They were maintained in the conventional animal facility of the University of Regensburg under standard conditions. Mice were anaesthetized with isoflurane, killed, and heart-perfused with PBS. Subsequent brains, lungs, hearts, spleens, and livers were harvested and snap frozen in liquid nitrogen immediately after removal.

### 4.3. Tissue Homogenization

Frozen material was homogenized in methanol/water (50/50, *v/v*), using a gentleMACS™ Dissociator (Miltenyi Biotec GmbH, Bergisch Gladbach, Germany), as described previously [13]. The homogenate was diluted in methanol/water (50/50, *v/v*) to a concentration of 0.05 mg/µL. Sample material was pooled tissue-wise to reduce inter-individual differences and to ensure sufficient sample material for all subsequent analyses. A total of 10% SDS solution was then added to the pooled tissue samples for the indicated final SDS concentrations. These samples were stored at room temperature for the indicated times and subsequently snap frozen in liquid nitrogen. Samples were stored at −80 °C until extraction.

### 4.4. Lipid Extraction

Samples were spiked with internal standards prior to lipid extraction (solvent of standards was removed by vacuum centrifugation). A detailed list of internal standards is found in Supplementary File Data S1. An amount of 2 mg wet weight was subjected to lipid extraction according to the protocol described by Bligh and Dyer [11] with a total chloroform volume of 2 mL. An amount of 1 mL (for FIA-MS/MS) and 0.5 mL (for FIA-FTMS) of the separated chloroform phase was transferred into sample vials by a pipetting robot (Tecan Genesis RSP 150, Männedorf, Switzerland) and vacuum dried. The residues were dissolved in either methanol/chloroform (3:1, *v/v*) with 7.5 mM ammonium acetate (FIA-MS/MS) or chloroform/methanol/2-propanol (1:2:4 *v/v*/*v*) with 7.5 mM ammonium formate (FIA-FTMS).

### 4.5. Lipid Analysis by Mass Spectrometry

The analysis of lipids was performed by direct flow injection analysis (FIA), using either a triple quadrupole mass spectrometer (FIA-MS/MS; QQQ triple quadrupole) or a hybrid quadrupole-Orbitrap mass spectrometer (FIA-FTMS; high mass resolution).

FIA-MS/MS (QQQ) was performed in positive ion mode using the analytical setup and strategy described previously [14,15]. A fragment ion of *m/z* 184 was used for PC and LPC [16]. The following neutral losses were applied: PE and LPE 141 [17]. PE-based plasmalogens (PE P) were analyzed according to the principles described by Zemski-Berry [18]. Sphingosine-based Cer were analyzed using a fragment ion of *m/z* 264 [19]. Quantification was performed with calibration lines, as previously described in detail [14].

A detailed description of the FIA-FTMS method was published recently [20,21]. TG, DG, and CE were recorded in positive ion mode as [M + NH_4_]^+^ in *m/z* range 500–1000 and a target resolution of 140,000 (at 200 *m/z*). CE were corrected for their species-specific response [22]. SM and ether PC (PC O) were analyzed as [M+HCOO]^-^ in negative ion mode in *m/z* range 520–960 at the same resolution setting. Multiplexed acquisition (MSX) was applied for the [M + NH_4_]^+^ of FC and the corresponding internal standard (D_7_-FC) [22]. Data processing details were described in Höring et al. [21], using the ALEX software [23] for peak assignment. Quantification was achieved by multiplication of the spiked-in IS amount with the analyte-to-IS intensity ratio. For both methods, lipid concentrations were referenced to the tissue wet weight (nmol/mg wet weight).

Lipid species were annotated according to the proposal for shorthand notation of lipid structures that are derived from mass spectrometry [1,24].

### 4.6. Evaluation Strategy

Concentration of lipid species was determined by *n* = 3 technical replicates. All lipid species displaying concentration >1% of total lipid composition at least in one sample were included in the data analysis. Values below the limit of quantitation (LOQ) were excluded from the data analysis. Liver samples were grouped according to their SDS concentration and heat map analyses of lipid species were performed, visualizing the relative increase or decrease of values, reported as log_2_-fold change compared to the control sample (0 h) of each group.

## 5. Conclusions

Physical, chemical, and/or enzymatic reactions influence the stability and detection of lipids. Lipid class ratios provide a simple but powerful readout to monitor the stability of samples and evaluate the feasibility of stabilizing agents or conditions. The addition of SDS quenches enzymatic activity and can be used to preserve lipid stability during sample storage and handling. Of note, stabilization is lipid class- and tissue-specific and needs to be evaluated respectively.

## Data Availability

Data are found in the Supplementary File Data S1.

## Supplementary Materials

The following are available online at https://www.mdpi.com/article/10.3390/metabo11050277/s1, Figure S1: FC/CE ratios of liver, brain, lung, heart, and spleen samples stored at room temperature for indicated times, Data S1: Lipid data.
