# Supplementary material for: Application of Lipid Class Ratios for Sample Stability Monitoring—Evaluation of Murine Tissue Homogenates and SDS as a Stabilizer
Source: Metabolites. 2021 Apr 27;11(5):277. doi: 10.3390/metabo11050277 (PMC8146515; doi:10.3390/metabo11050277)
Supplement: Supplementary file 1 [file metabolites-11-00277-s001.zip › metabolites-1176017-supplementary/Figure S1.pdf]

metabolites

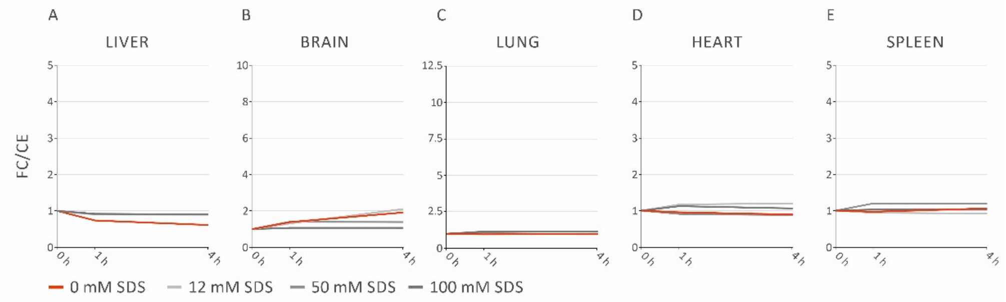

Figure S1. FC to CE ratios of liver, brain, lung, heart and spleen samples stored at room temperature for indicated times. The fold changes of the ratios are displayed relative to the control sample (0 h). CE: cholesteryl ester; FC: free cholesterol; SDS: sodium dodecyl sulfate.
